# rWTC‐MBTA Vaccine, Alone and Enhanced with Anti‐PD1, Elicits Immune Responses against CNS and Peripheral B‐Cell Lymphoma

**DOI:** 10.1002/advs.202511605

**Published:** 2025-12-08

**Authors:** Yaping Zhang, Juan Ye, Mitchell Sun, Samik Chakraborty, Alex Valenzuela, Shuran Chen, Qingfeng Xue, Wenyu Shi, Karel Pacak, Herui Wang, Zhengping Zhuang

**Affiliations:** ^1^ Neuro‐Oncology Branch Center for Cancer Research National Cancer Institute National Institutes of Health Bethesda MD 20892 USA; ^2^ Department of Hematology Affiliated Hospital of Nantong University 20 Xisi Road Nantong Jiangsu 226001 China; ^3^ NE1 Inc. New York NY 10022 USA; ^4^ David Geffen School of Medicine University of California Los Angeles CA 90095 USA; ^5^ Department of Oncology Affiliated Hospital of Nantong University 20 Xisi Road Nantong Jiangsu 226001 China; ^6^ Eunice Kennedy Shriver National Institute of Child Health and Human Development National Institutes of Health 9000 Rockville Pike Bethesda MD 20892 USA; ^7^ AKESO Center for Adrenal Endocrine Tumors Prague 5 Prague 15000 Czech Republic

**Keywords:** anti‐PD1 therapy, CNS lymphoma, Immune memory, rWTC‐MBTA vaccine, tumor microenvironment

## Abstract

Central nervous system (CNS) lymphomas are difficult to treat due to their aggressive nature, limited brain accessibility, and poor response to conventional therapies. This study evaluates the rWTC‐MBTA vaccine, alone or with anti‐PD1 therapy, in A20 B‐cell lymphoma models. The vaccine alone significantly inhibits tumor growth and extends survival in both subcutaneous and intracranial settings by triggering strong innate and adaptive immune responses, including long‐term memory. Immune profiling reveals dynamic responses in lymph nodes and tumor‐infiltrating lymphocytes over time. Co‐culture assays with lymph node or spleen cells from vaccinated mice show enhanced tumor cell killing and increased IFN‐γ and TNF‐α levels, indicating lymphoma‐specific immunity. In the intracranial model, control mice has dense tumor infiltration in the brain, while vaccinated mice exhibit more dispersed tumors with reduced spread to ventricles and meninges. Vaccination also shifts the tumor microenvironment toward a more active antitumor state, marked by increased PD‐1⁺ CD8⁺ T cells. Combining the vaccine with anti‐PD1 further enhances antitumor effects. These findings demonstrate that rWTC‐MBTA induces potent and durable immune responses against CNS and peripheral lymphomas, offering long‐term protection and showing synergistic benefit when combined with immune checkpoint blockade.

## Graphical abstract

An autologous whole‐tumor‐cell vaccine (rWTC‐MBTA) is evaluated in murine CNS lymphoma. Subcutaneous vaccination activates dendritic cells, broadens T‐cell priming, and drives lymphocyte trafficking to brain tumors, producing durable tumor control. Longitudinal bioluminescence and adoptive‐transfer assays verify CNS engagement. Combination with anti‐PD‐1 further prolongs survival and prevents relapse, supporting clinical translation.

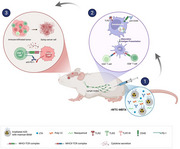

## 1 Introduction

Lymphoma, a malignancy of the lymphatic system, exhibits a wide range of prognoses depending on its type and stage. Despite therapeutic advances, aggressive forms of lymphoma remain challenging, with more than half of patients experiencing relapse and limited responses to subsequent treatments.^[^ ^1^ ^]^ Lymphomas that arise in immune‐privileged sites, such as the central nervous system (CNS), are particularly difficult to treat due to poor drug penetration. Most CNS lymphomas (CNSLs) are diffuse large B‐cell lymphomas (DLBCL), one of the most aggressive subtypes of non‐Hodgkin lymphoma. Although CNSL is potentially curable, the 5‐year survival rate for primary CNSL remains low at 30–40%.^[^ ^2^ ^]^ Secondary CNSL often arises within months of the initial lymphoma diagnosis and is associated with especially poor outcomes and a median survival of just 6 months.^[^ ^2^, ^3^, ^4^ ^]^ As such, there is growing emphasis on improving treatment strategies for primary CNSL while developing effective prophylactic approaches for secondary CNSL.

Immunotherapy has revolutionized the treatment of B‐cell lymphoma by harnessing the immune system to combat malignancies. Monoclonal antibodies (mAbs), such as rituximab, target the CD20 protein on B cells,^[^ ^5^ ^]^ while chimeric antigen receptor (CAR) T‐cell therapy provides a personalized strategy by genetically engineering T cells to recognize and eliminate lymphoma cells.^[^ ^6^ ^]^ Similarly, bispecific antibodies (BsAbs) facilitate T‐cell‐mediated cytotoxicity by simultaneously binding tumor antigens and CD3 on T cells.^[^ ^7^, ^8^ ^]^ These therapies have significantly expanded treatment options and enabled durable remissions in many patients. However, challenges such as immune escape and antigen loss underscore the need for more resilient and broadly effective therapeutic strategies.^[^ ^6^, ^9^, ^10^ ^]^

Cancer vaccines represent a significant advancement in oncology, offering a therapeutic strategy that harnesses systemic immune mechanisms to recognize and eliminate malignant cells through broad‐spectrum antigen recognition.^[^ ^11^, ^12^ ^]^ Unlike conventional therapies, these vaccines have the potential to induce durable immune memory and long‐term protection against tumor recurrence, enabling sustained clinical remissions.^[^ ^11^, ^12^ ^]^ Among these approaches, autologous tumor cell‐based vaccines (ATVs), which present a wide array of patient‐specific neoantigens, are particularly promising. However, ATVs have shown limited clinical efficacy across multiple cancer types.^[^ ^13^, ^14^, ^15^, ^16^ ^]^ Clinical candidates such as Vigil and GVAX have generally elicited weak immune responses and modest therapeutic benefit, largely due to low neoantigen immunogenicity, tumor heterogeneity, immunosuppressive tumor microenvironments (TMEs), and suboptimal antigen processing and presentation.^[^ ^11^, ^12^, ^13^, ^14^, ^15^, ^16^, ^17^ ^]^

To address these limitations, we developed rWTC‐MBTA, a next‐generation autologous tumor cell‐based vaccine designed to overcome immune suppression, enhance antigen presentation, and elicit durable anti‐tumor immunity.^[^ ^18^, ^19^, ^20^ ^]^ rWTC‐MBTA works by inserting Mannan‐BAM into the membranes of irradiated tumor cells to mimic pathogen‐associated molecular patterns (PAMPs) for enhancing immune recognition.^[^ ^18^ ^]^ The co‐delivery of multiple TLR ligands (Resiquimod, poly I:C, and LTA) and an anti‐CD40 antibody helps stimulate antigen‐presenting cells like dendritic cells and macrophages to prime tumor‐specific T cell responses.^[^ ^20^ ^]^ This integrated adjuvant strategy initiates robust innate and subsequent adaptive immunity. Prior studies in metastatic breast tumor and glioblastoma preclinical models demonstrated that rWTC‐MBTA dynamically activates dendritic cells, enhances T cell responses, and improves survival outcomes.^[^ ^18^, ^20^, ^21^ ^]^

Building on this foundation, we developed the rWTC‐MBTA vaccine using irradiated A20 cells, aiming to evaluate the efficacy of rWTC‐MBTA in B‐cell lymphoma models, including CNSL. We hypothesize that this strategy eliciting activation of both innate and adaptive immunity will trigger strong and long‐lasting lymphoma‐specific immune responses and overcome the unique immunosuppressive environment of CNSL. Moreover, we hypothesize that rWTC‐MBTA will synergize with immune checkpoint inhibitors by enhancing T cell activation and tumor infiltration, thereby addressing some key limitations of current immunotherapies.

## 2 Results

### 2.1 rWTC‐MBTA Vaccine Prolongs Survival and Induces Long‐Term Immune Memory in a Subcutaneous A20‐Lymphoma Model

**Figure 1:**
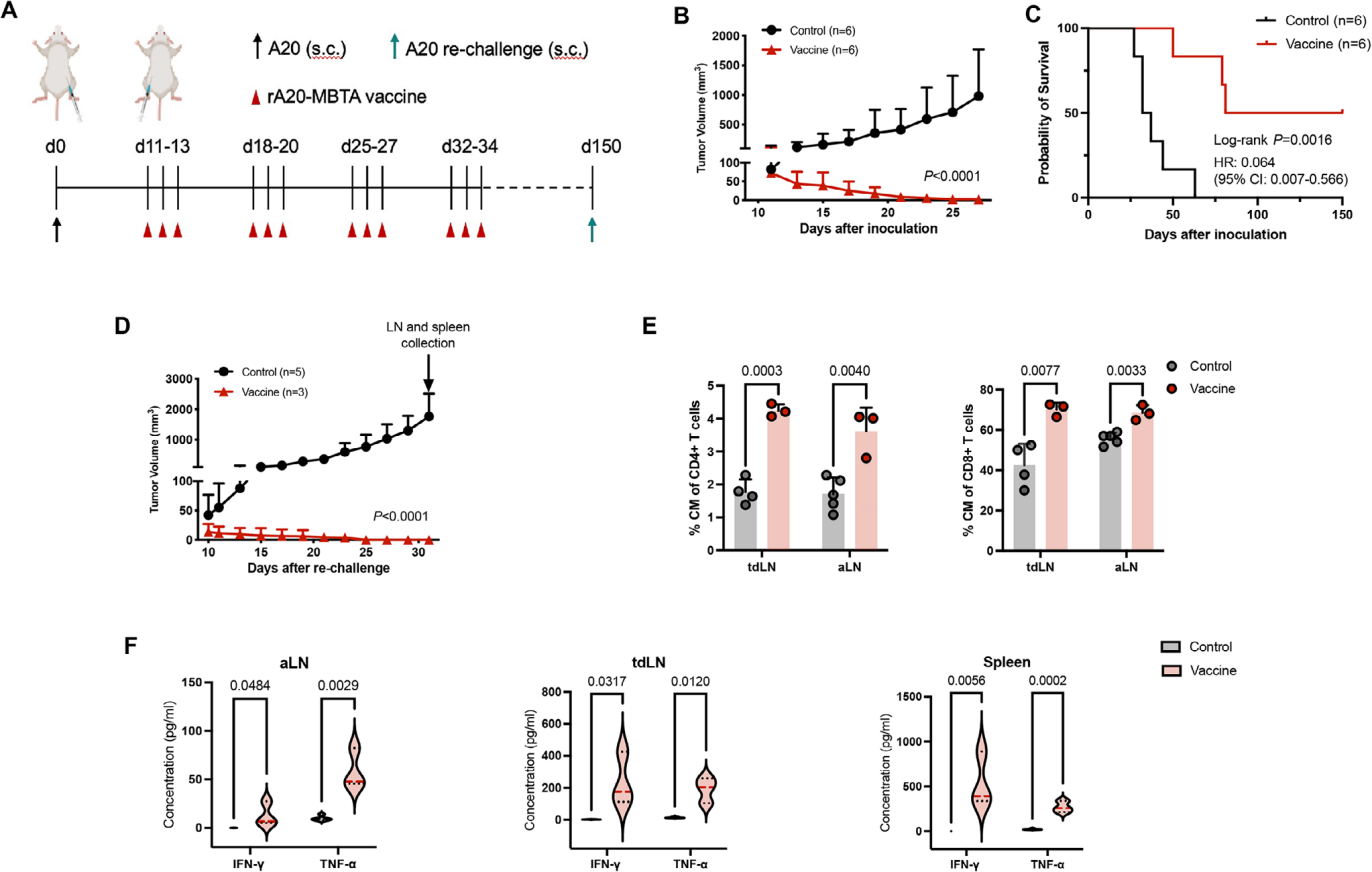
rWTC‐MBTA vaccine prolonged survival and induced long‐term immune memory in subcutaneous A20 lymphoma mice. A) Diagram depicting the treatment regimen for mice with A20 lymphoma. BALB/c mice were injected subcutaneously with 1 million A20 cells into the right flank. Eleven days following the inoculation of tumor cells, treatments (control and rWTC‐MBTA vaccine) were injected subcutaneously into the left flank region. Treatments were given over three consecutive days and continued weekly for four weeks. B) Tumor growth curves showing average tumor volume over time for the two groups (n = 6 per group). The mean tumor volumes are plotted, with error bars representing the standard deviation (SD). *p *< 0.0001, One‐way ANOVA. C) Kaplan–Meier survival curves across the two groups (n = 6 per group). *p *= 0.0016, Log‐rank (Mantel–Cox) test. Hazard ratios with 95% confidence intervals (CI) are also shown. D) Tumor growth curves after A20 rechallenge. Data shown as mean tumor volume ± SD over time. E) Bar graphs depict the percentage of central memory T cells within CD4⁺ and CD8⁺ T cells from tdLN and aLN, showing higher frequencies in rWTC‐MBTA–vaccinated mice versus controls. tdLN, tumor‐draining lymph nodes; aLN, axillary lymph nodes. F) Co‐culture assays with immune cells from tdLN, aLN and spleen demonstrate that the rWTC‐MBTA vaccine prompts a more potent release of the cytokines IFN‐γ and TNF‐α. *p* values from unpaired T‐tests are shown.

To assess the therapeutic potential of the rWTC‐MBTA vaccine in lymphomas, we established a subcutaneous A20 lymphoma model in BALB/c mice. Mice were inoculated with 1 million A20 cells into the right flank. Once tumors reached an average volume of 60 mm^3^ (≈11 days post‐inoculation), mice were randomized into two cohorts: a control group (PBS‐treated) and an rWTC‐MBTA vaccine group. The rWTC‐MBTA vaccine, prepared from irradiated A20 lymphoma cells, was administered subcutaneously into the left flank for three consecutive days per week over a four‐week regimen (**Figure** **1**A). Treatment with rWTC‐MBTA significantly inhibited tumor progression compared to controls (*p* < 0.0001; Figure 1B). Vaccinated mice exhibited significantly extended survival time until study endpoint compared to the control group (*p *= 0.0016; Figure 1C). Notably, 50% of vaccinated mice (3 out of 6 mice) achieved complete tumor regression (CR).

To evaluate the establishment of long‐term immune memory, CR mice were rechallenged with A20 cells 150 days after the initial tumor implantation. All rechallenged CR mice remained tumor‐free, whereas all age‐matched control mice developed lymphoma (*p* < 0.0001; Figure 1D). On day 31 post‐rechallenge, when two control mice reached study endpoint criteria, all animals were sacrificed for immunological analysis. Compared to the control group, vaccinated mice showed increased frequencies of central memory (CM) T cells in both CD4⁺ and CD8⁺ T cell populations in the tumor‐draining lymph nodes (tdLN), axillary lymph nodes (aLN), and spleen (Figure 1E). Ex vivo co‐culture of these immune cells with A20 cells (10:1 ratio) revealed significantly elevated secretion of IFN‐γ and TNF‐α in the vaccine group compared with the control group, reflecting enhanced tumor‐specific T cell responses (Figure 1F).

Together, these results demonstrate that rWTC‐MBTA not only induces strong primary antitumor immunity but also establishes durable immune memory that protects against lymphoma recurrence.

### 2.2 rWTC‐MBTA Vaccine Induces Dynamic Dendritic Cell Responses

To investigate the mechanisms underlying rWTC‐MBTA‐induced antitumor immunity, we analyzed innate and adaptive immune responses in lymph nodes at two time points: one day after the first vaccine cycle (initial three consecutive doses) and one day after the second cycle. All DC metrics are reported as a percentage of total live cells in the vaccine‐draining lymph node (injection‐site LN, isLN).

**Figure 2:**
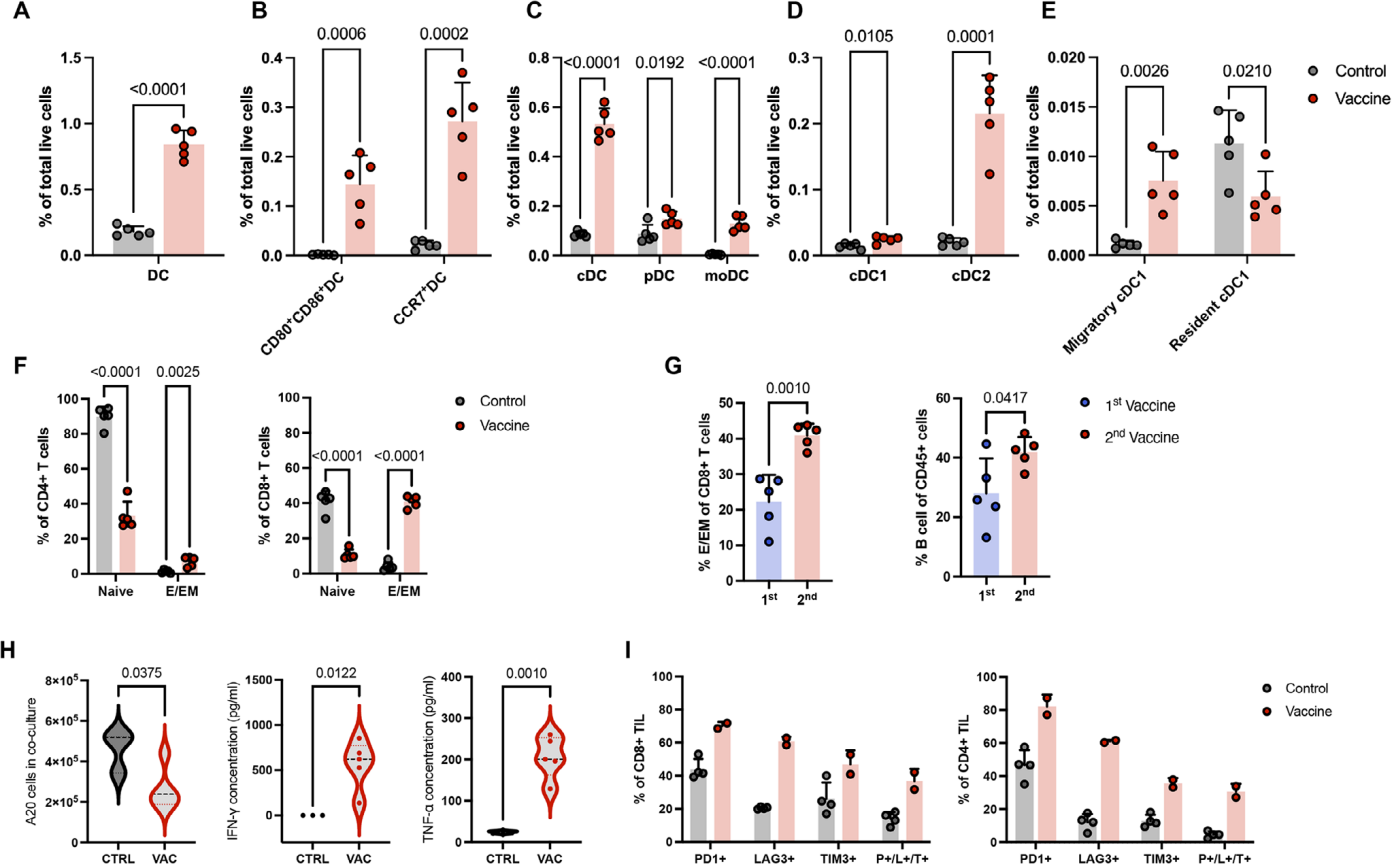
rWTC‐MBTA vaccine induces dynamic dendritic cell responses, enhances adaptive immunity, and upregulates T‐cell checkpoint molecules. A–E) Percentage of total DC (A) and DC subset in total live cells after the first vaccination cycle. F) Distribution of naïve T cells and effector and/or effector memory (E/EM) T cells within CD4^+^ and CD8^+^ T cells. G) After the second vaccine cycle, there was an increase in CD8+ E/EM T cells, as well as B cells, within the isLN. N = 5 for each group; unpaired T‐test. H) Co‐cultures from the vaccine group (n = 5) displayed fewer A20 tumor cells compared to the control group (n = 3). Supernatants from the vaccine group showed increased concentrations of IFN‐γ and TNF‐α cytokines. I) Flow cytometry after the second vaccine cycle showed significant increases in PD‐1, LAG‐3, TIM‐3, and their co‐expression in CD4^+^ and CD8^+^ T cells within the TILs of the vaccine group.

Dendritic cells (DCs) are central to bridging innate and adaptive immunity and have a flexible phenotype that is shaped by environmental cues. Total DC abundance increased after both cycles compared to controls (cycle 1: 0.84% vs 0.18%; cycle 2: 0.51% vs 0.14%) (**Figure** **2**A; Figure S1A, Supporting Information). Following the first vaccination cycle, DCs displayed robust maturation/trafficking, with higher CD80⁺CD86⁺ DCs (0.144% vs 0.0017%) and CCR7⁺ DCs (0.270% vs 0.021%; Figure 2B). This was accompanied by increased conventional DCs (cDCs, 0.532% vs 0.087%) and monocyte‐derived DCs (moDCs, 0.130% vs 0.0046%), with a modest pDC increase (0.149% vs 0.088%) (Figure 2C). Within cDCs, the response was cDC2‐dominant after the first vaccination cycle (cDC2: 0.220% vs 0.020%; cDC1: 0.0244% vs 0.0145%), accompanied by enrichment of migratory cDC1 (Figure 2D,E).

After the second vaccination cycle, **CD80⁺CD86⁺ DCs** remained above control (**0.0158% vs 0.0059%**) with a trend toward increased **CCR7⁺ DCs** (**0.040% vs 0.027%**), and the total DC population remained elevated (Figure S1A,B, Supporting Information). cDCs, moDCs, and pDCs remained significantly higher than the controls (Figure S1C, Supporting Information). Notably, the cDC composition shifted toward cDC1, which increased markedly (0.0927% vs 0.0119%), whereas cDC2 increased more modestly (0.0539% vs 0.0148%) (Figure S1D, Supporting Information). Concomitantly, resident cDC1 increased as migratory cDC1 contracted (Figure S1E, Supporting Information), consistent with a transition from antigen transport to sustained local cross‐presentation.

Collectively, these findings highlight the temporal plasticity and activation dynamics of DC subsets following rWTC‐MBTA vaccination in the A20 lymphoma models.

### 2.3 rWTC‐MBTA Vaccine Enhances Adaptive Immune Responses while Inducing Expression of Checkpoint Proteins in Subcutaneous Lymphoma Models

We next examined the dynamic changes in adaptive immune responses within lymph nodes following the first and second rWTC‐MBTA vaccine treatment cycles, with a particular focus on responses after the second cycle. In the control group, naïve T cells (CD44^−^/CD62L⁺) predominated among both CD4⁺ and CD8⁺ subsets. In contrast, the vaccine group exhibited a significant expansion of effector and/or effector memory (E/EM) T cells (CD44⁺/CD62L^−^) within both CD4⁺ and CD8⁺ populations after the second vaccination (Figure 2F). During sequential immunizations, a marked increase in E/EM CD8⁺ T cells and B cells was observed in lymph nodes following the second vaccination, whereas E/EM CD4⁺ T cell frequencies remained largely unchanged (Figure 2G; Figure S2, Supporting Information).

To assess tumor‐specific adaptive immunity, immune cells from the injection‐site lymph nodes (isLNs) of vaccinated mice were co‐cultured with A20 tumor cells. After 48 h, the vaccine group showed a significantly smaller number of viable A20 cells compared to the control group (Figure 2H). This cytotoxic activity was accompanied by markedly elevated levels of IFN‐γ and TNF‐α in the co‐culture supernatants, indicating an enhanced antigen‐specific cytotoxic response upon tumor re‐exposure (Figure 2H). These findings demonstrate that the rWTC‐MBTA vaccine elicits robust, tumor‐specific adaptive immune responses.

To determine whether the antitumor efficacy of the rWTC‐MBTA vaccine is T cell–dependent, we performed CD4⁺, CD8⁺, and combined CD4⁺/CD8⁺ T cell depletion experiments. Effective depletion was confirmed by flow cytometry analysis of peripheral blood (Figure S3A, Supporting Information). In vaccinated mice lacking both CD4⁺ and CD8⁺ T cells, A20 lymphoma growth was significantly accelerated and comparable to that observed in the control group. Among single T cell‐depleted groups, CD8⁺ T cell depletion led to more rapid tumor progression, while CD4⁺ T cell depletion resulted in a modest reduction in vaccine efficacy compared to the intact vaccine group (Figure S3B, Supporting Information). Survival analysis further demonstrated a substantial decline in survival in mice depleted of CD8⁺ or both CD4⁺ and CD8⁺ T cells. In contrast, CD4⁺ T cell‐depleted mice exhibited survival rates similar to the intact vaccine group, with about 50% of the mice reaching CR (Figure S3C, Supporting Information). These findings confirm that the rWTC‐MBTA vaccine relies on both CD4⁺ and CD8⁺ T cells for its efficacy in the A20 lymphoma model, with CD8⁺ T cells playing a dominant role in mediating antitumor protection.

We further assessed the expression of immune checkpoint markers associated with T cell exhaustion, including PD‐1, LAG‐3, and TIM‐3, on tumor‐infiltrating lymphocytes (TILs). Following the second vaccination cycle, the rWTC‐MBTA vaccine group exhibited significantly higher expression levels of PD‐1, LAG‐3, and TIM‐3 on both CD4⁺ and CD8⁺ T cells compared to the control group (Figure 2I). No significant changes in the frequencies of PD‐1⁺, LAG‐3⁺, or TIM‐3⁺ T cells were observed between the first and second immunizations within the vaccine group (Figure S4, Supporting Information).

In addition, the proportion of regulatory T cells (Tregs) within the CD4⁺ population in the tumor microenvironment was elevated in the vaccine group relative to controls (Figure S5A, Supporting Information). Increased Treg frequencies were also observed in peripheral lymphoid organs, particularly the spleen (Figure S5B,C, Supporting Information). These findings suggest that, while the rWTC‐MBTA vaccine induces robust T cell responses, it may also trigger compensatory counter‐regulation, namely increased T cell checkpoint expression and Treg expansion, highlighting actionable targets to further enhance the vaccine efficacy.

### 2.4 rWTC‐MBTA Vaccine Demonstrates Therapeutic Efficacy and Dynamic Immune Modulation in a CNS Lymphoma Model

To evaluate the therapeutic efficacy of the rWTC‐MBTA vaccine in CNS lymphoma, we established a murine model via intracranial injection of 50 000 A20 lymphoma cells into the right frontal lobe of BALB/c mice. Brain histology confirmed successful tumor implantation five days post‐injection (Figure S6, Supporting Information).

**Figure 3:**
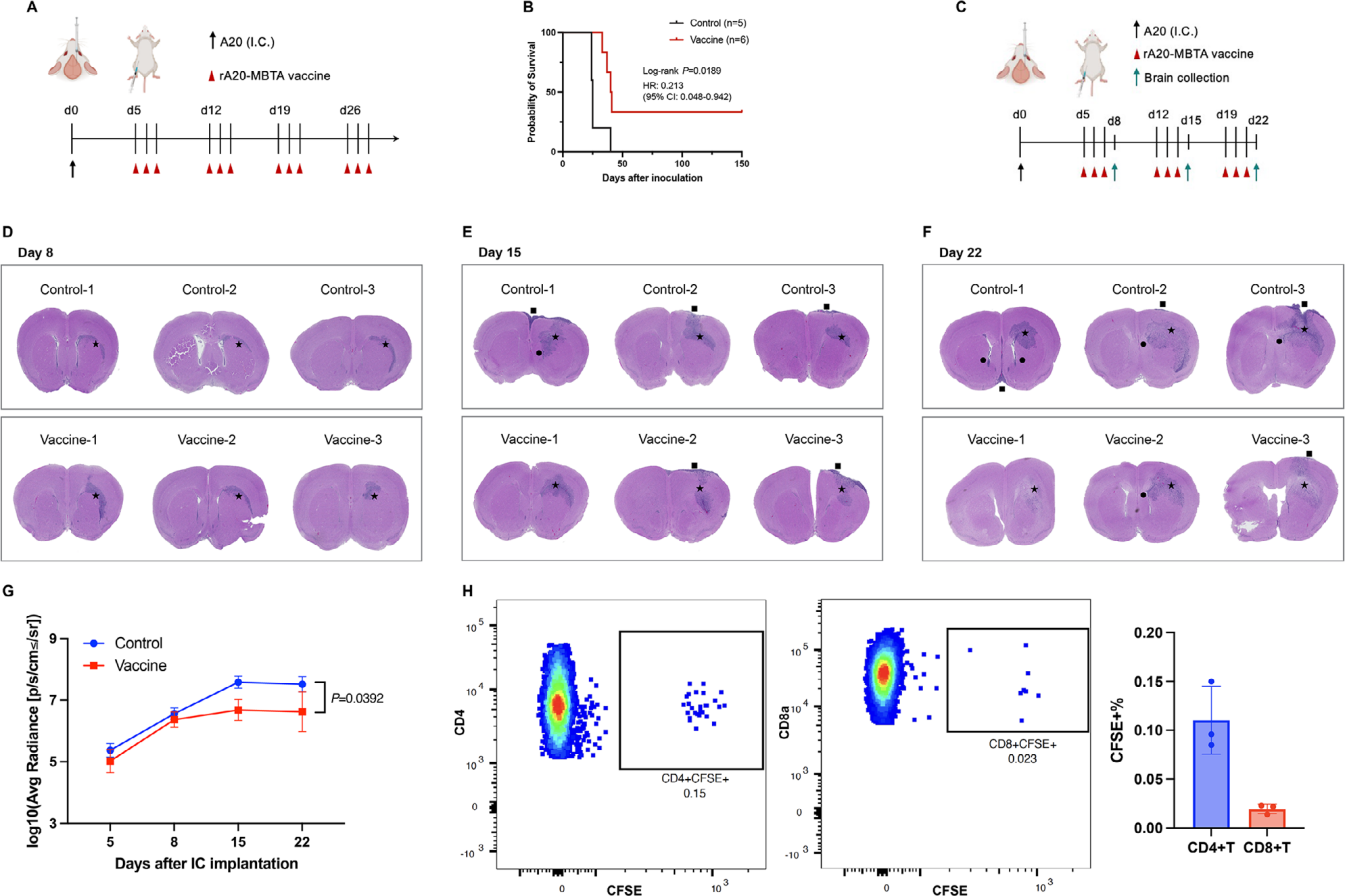
rWTC‐MBTA vaccine demonstrates therapeutic efficacy in a CNS lymphoma model. A) Diagram of rWTC‐MBTA vaccine administration in the CNS lymphoma model. The A20 CNS lymphoma model was initiated with an intracranial injection of 50 000 A20 cells. Starting five days after cell implantation, mice received subcutaneous injections of either PBS (control) or the rWTC‐MBTA vaccine into the left flank, following a regimen of three consecutive injections per week over four weeks. B) Kaplan–Meier curves illustrate the survival benefit conferred by the rWTC‐MBTA vaccine. *p *= 0.0189, Log‐rank test. Hazard ratios with 95% CI are also shown. C) Schematic of rWTC‐MBTA vaccine administration and brain collection. D–F) HE staining illustrated brain tissue architecture and tumor regions on Day 8 (D), Day 15 (E), and Day 22 (F) following intracranial A20 cell inoculation. Symbolic annotations demonstrate distinct invasion patterns: ★ (parenchymal invasion), ■ (meningeal infiltration), and ● (lateral ventricular involvement). N = 3 per group. G) Longitudinal bioluminescence imaging (BLI) of B‐luc–A20 CNS lymphomas at the indicated time points. N = 7 for the control group, and N = 12 for the vaccine group. Data shown as mean ± SEM. Two‐way ANOVA (treatment effect, control vs vaccine): *p* = 0.0392. H) CFSE‐labeled CD4^+^ and CD8^+^ T cells were confirmed in the mouse brain after adoptive immune cell transfer. N = 3 for the recipient mice.

Mice were then randomized into two groups: a control group and an rWTC‐MBTA vaccine group. Vaccination began five days after tumor implantation and was administered subcutaneously to the left flank for three consecutive days per week over four weeks (**Figure** **3**A). Compared to the control group, rWTC‐MBTA treatment significantly prolonged survival (median survival: 40.5 vs 25 days, *p* = 0.0189, Log‐rank test), with 33.3% of vaccinated mice (2 of 6) achieving CR (Figure 3B). These findings suggest the strong therapeutic potential of rWTC‐MBTA in CNS lymphoma.

To evaluate the dynamic efficacy of the rWTC‐MBTA vaccine, brain tissues were collected at Days 8, 15, and 22—corresponding to one day after each of the first three vaccination cycles—for histopathological analysis (Figure 3C). On Day 8, H&E staining revealed similar tumor growth patterns in both the control and vaccine groups, with tumors forming clusters within the parenchyma (Figure 3D). By Day 15, tumors expanded in both groups and began infiltrating the meninges (Figure 3E). By Day 22, 17 days after initial vaccination, control group tumors showed extensive invasion into the parenchyma, meninges, and lateral ventricles, while maintaining a clustered morphology. In contrast, the vaccine group exhibited low‐cell‐density and dispersed tumor cells limited to the parenchyma and meninges, with less meningeal and ventricular involvement (Figure 3F).

We also performed a longitudinal imaging study using a luciferase‐labeled A20 line (B‐luc–A20). We intracranially implanted 1 × 10⁵ B‐luc‐A20 cells and began vaccination on Day 5, identical to our prior CNS A20 lymphoma model. Bioluminescence imaging (BLI) at standardized time points showed no clear group difference on Day 8 (one day after the first vaccination cycle), with reduced photon flux in the vaccine group on Days 15 and 22 (Figure 3G, two‐way ANOVA test, treatment effect: *p *= 0.0392). These kinetics are concordant with the extended survival and histopathological findings.

To directly demonstrate CNS entry of vaccine‐primed cells, we isolated injection‐site lymph nodes (isLN) after three weeks of vaccination, labeled dissociated cells with CFSE, and adoptively transferred them intravenously into B‐luc‐A20 CNS lymphoma–bearing recipient mice (about 2 × 10⁶ isLN cells per mouse). After 3 days, mice were transcardially perfused with PBS to remove intravascular cells, brains were processed to isolate brain‐infiltrating leukocytes, and samples were analyzed by flow cytometry.^[^ ^22^ ^]^ CFSE⁺ cells were detected among brain‐infiltrating T cells, accounting for 0.11% of CD4⁺ and 0.02% of CD8⁺ T cells, confirming that peripherally primed lymphocytes can traverse CNS barriers and enter the brain tissue (Figure 3H).

**Figure 4:**
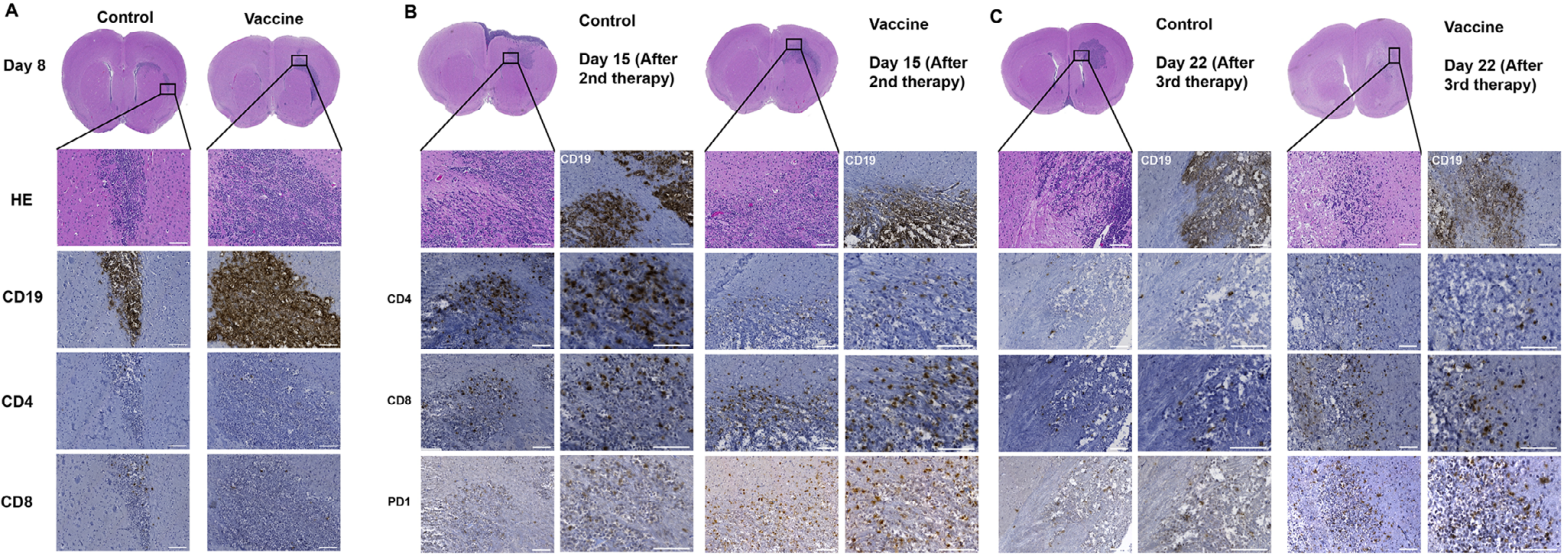
rWTC‐MBTA vaccine demonstrates dynamic immune modulation in a CNS lymphoma model. Histopathological and immunohistochemical analyses were performed to assess tumor microenvironment changes. HE staining reveals the architecture of brain tissue and tumor morphology, while immunohistochemistry (IHC) for CD19 highlights areas occupied by A20 B cell lymphoma. A) Representative HE staining and CD19, CD4, and CD8 staining in control and vaccinated mice on Day 8 (one day after the first vaccine cycle). B,C) HE staining and CD19, CD4, CD8, and PD‐1 staining in control and vaccinated mice on Day 15 (one day after the second vaccine cycle, B) and Day 22 (one day after the third vaccine cycle). Representative images from three mice per group are shown at each time point. Right‐hand panels show higher‐magnification views of CD4, CD8, and PD‐1 staining. Scale bars = 100 µm.

Immunohistochemistry (IHC) staining further examined the tumor immune microenvironment (**Figure** **4**). CD19 staining was used to identify lymphoma regions. On Day 8, CD4⁺ and CD8⁺ T cell infiltration was minimal and comparable between the control and vaccine groups (Figure 4A). By Day 15, both groups showed increased T cell infiltration, but the vaccine group had a notable rise in CD8⁺ T cells, while CD4⁺ T cells predominated in controls. PD‐1 expression was also higher in the vaccine group, consistent with findings in the subcutaneous lymphoma models (Figures 4B and 2I). On Day 22, CD4⁺ and CD8⁺ T cells declined in the control group. In contrast, the vaccine group maintained high CD8⁺ T cell infiltration and PD‐1 expression, with low CD4⁺ T cell presence (Figure 4C).

Together, these results demonstrate that rWTC‐MBTA vaccination elicits a sustained and dynamic antitumor immune response in CNS lymphoma, characterized by increased CD8⁺ T cell infiltration and altered tumor histopathology.

### 2.5 Anti‐PD1 Enhanced rWTC‐MBTA Vaccine Immune Response while Prolonging Survival and Preventing Long‐Term Recurrence

**Figure 5:**
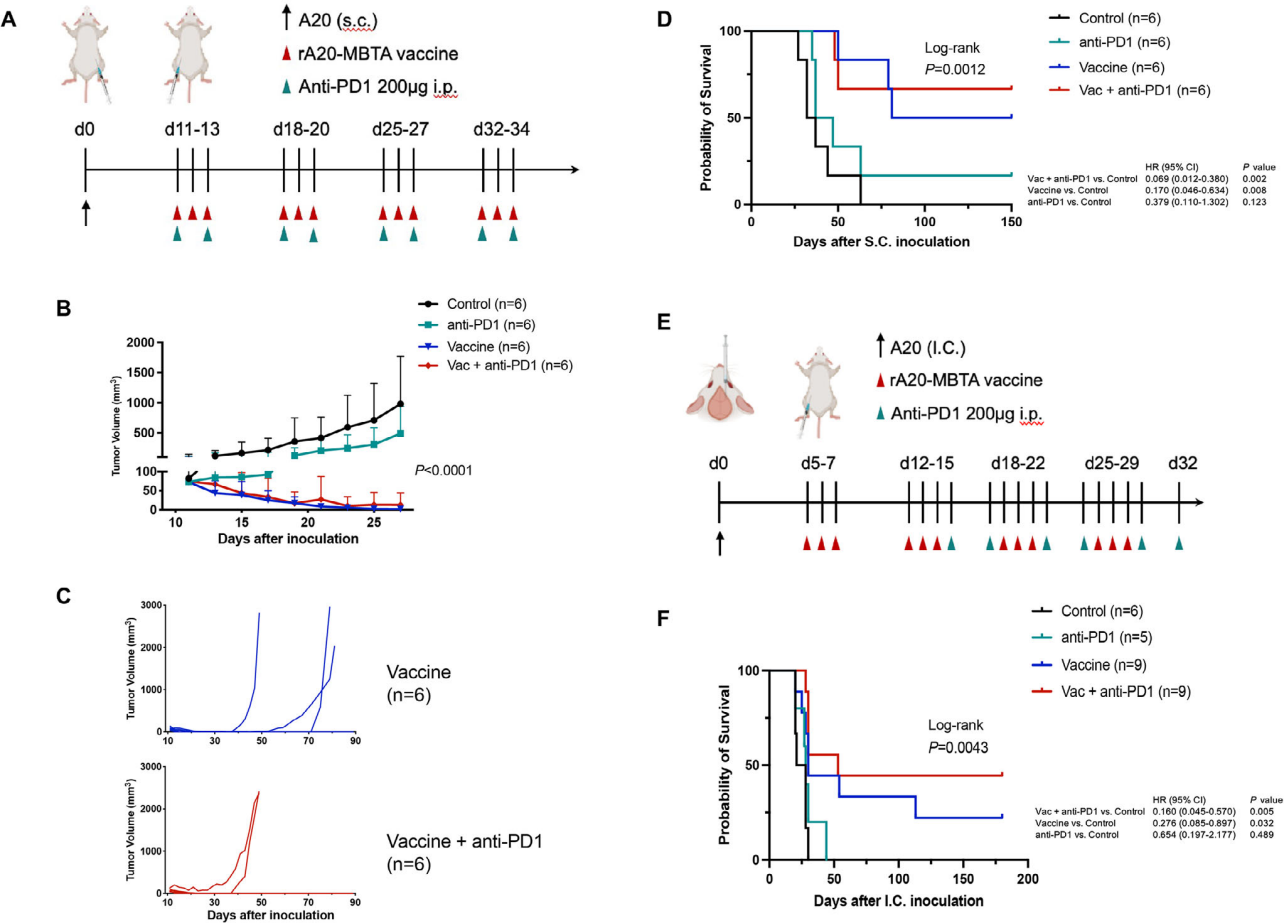
Anti‐PD1 enhances rWTC‐MBTA vaccine responses, prolongs survival, and prevents long‐term recurrence in subcutaneous and intracranial A20 lymphoma models. A) Schematic of rWTC‐MBTA vaccine and anti‐PD1 administration in the subcutaneous A20 lymphoma model. B) Tumor growth curves showing average tumor volume over time in the four groups (n = 6 per group). *p *< 0.0001, One‐way ANOVA. C) Individual tumor growth trajectories for each mouse in the subcutaneous lymphoma model across the vaccine group and combined group. D) Kaplan–Meier survival curves for the four groups in the subcutaneous A20 lymphoma model. (n = 6 per group). *p *= 0.0012, Log‐rank (Mantel‐Cox) test. *p*‐values are shown for comparisons between all groups. Hazard ratios with 95% CI are also shown. E) Schematic of rWTC‐MBTA vaccine and anti‐PD1 administration in the intracranial A20 lymphoma model. F) Kaplan–Meier survival curves for the four treatment groups. *p *= 0.0043, Log‐rank (Mantel‐Cox) test. *p*‐values are shown for comparisons between all groups. Hazard ratios with 95% CI are also shown.

The elevated expression of PD‐1 observed in both peripheral and CNS lymphoma models suggests its potential as a therapeutic target to enhance rWTC‐MBTA vaccine efficacy. To investigate this, we evaluated the combination of the rWTC‐MBTA vaccine with anti‐PD‐1 therapy in a subcutaneous A20 lymphoma model. On Day 11 post‐inoculation, mice were randomized into four groups: Control (PBS), anti‐PD‐1 alone, rWTC‐MBTA vaccine alone, and the combination of rWTC‐MBTA vaccine with anti‐PD‐1. The same vaccination regimen began on Day 11, and anti‐PD‐1 antibodies were administered intraperitoneally twice a week for four weeks, aligned with the vaccination schedule on the first and third days of each cycle (**Figure** **5**A).

Tumor growth was significantly slower in both the rWTC‐MBTA and combination groups compared to the control and anti‐PD‐1 groups (Figure 5B). In the vaccine‐only group, however, two mice experienced tumor relapse after initially achieving complete response (CR): one achieved CR by Day 13 but relapsed on Day 39, and the other achieved CR by Day 15 but relapsed on Day 75, suggesting potential immune escape and residual tumor resistance. In contrast, no recurrences were observed in the combination group (Figure 5C). Survival analysis demonstrated that the combination group had the highest survival rate (*P* = 0.0012). CR was achieved in 50% (3/6) of mice in the vaccine‐only group and 66.7% (4 of 6) in the combination group (Figure 5D).

To assess the therapeutic potential of this combination in CNS lymphoma, we performed a parallel study using an intracranial A20 lymphoma model. Anti‐PD‐1 treatment began on Day 15 post‐inoculation and was administered intraperitoneally five additional times in coordination with the vaccine schedule—one day before and after each vaccination cycle (Figure 5E). The combination group again demonstrated the highest survival rate (44.4%, 4 of 9), compared to 22.2% (2 of 9) in the vaccine‐only group. No CR was observed in the control or anti‐PD‐1 monotherapy groups. Most animals reached the study endpoint around Day 30, with extended survival observed in one combination‐treated mouse (Day 53) and two vaccine‐treated mice (Days 54 and 113) (Figure 5F).

Collectively, these results demonstrate that combining the rWTC‐MBTA vaccine with anti‐PD‐1 therapy improves survival outcomes and reduces tumor recurrence in B‐cell lymphoma models, supporting this strategy as a promising therapeutic approach.

## 3 Discussion

Central nervous system lymphomas (CNSLs), including both primary (PCNSL) and secondary (SCNSL) forms, represent some of the most aggressive and treatment‐refractory hematologic malignancies. Over 90% of cases exhibit diffuse large B‐cell lymphoma (DLBCL) histology, a subtype associated with high proliferative indices and significant genomic instability.^[^ ^2^, ^3^, ^23^, ^24^ ^]^ Despite advances in high‐dose methotrexate‐based chemotherapy and autologous stem cell transplantation, outcomes remain dismal—especially in SCNSL, which involves systemic spread to the CNS. Median post‐relapse survival is only 6.8 months in PCNSL and as low as 3.9 months in SCNSL, highlighting the urgent need for new therapeutic approaches.^[^ ^2^, ^4^, ^23^, ^25^ ^]^

A key barrier to treatment success is the blood‐brain barrier (BBB), which restricts the delivery of large or hydrophilic agents into the CNS, requiring high systemic dosing and incurring significant toxicity.^[^ ^24^, ^26^, ^27^ ^]^ While radiation can transiently disrupt the BBB, its neurotoxicity limits repeated or long‐term use. Immunotherapy has revolutionized lymphoma treatment, but its application in CNSL remains limited. For example, although CAR T‐cell therapy has shown promise in systemic DLBCL, its efficacy in CNSL is reduced. A systematic review of 12 studies involving 69 patients reported a pooled relapse rate of 45%.^[^ ^28^ ^]^ In a retrospective study of 90 SCNSL patients treated with CD19‐directed CAR T‐cells, 50% achieved complete CNS remission at 3 months, but only 16% remained progression‐free at two years, and the overall survival was 31%.^[^ ^29^ ^]^ These results underscore the difficulty of sustaining CAR T‐cell function in the immunosuppressive and antigenically diverse CNS tumor microenvironment (TME).^[^ ^25^ ^]^

Tumor vaccines offer an alternative immunotherapeutic approach, capable of priming *de novo* tumor‐specific immune responses and establishing immune memory. This long‐term surveillance can detect and eliminate residual tumor cells, preventing relapse and improving durable remission rates.^[^ ^14^ ^]^ In our study, we evaluated the efficacy of the rWTC‐MBTA vaccine—a whole tumor cell‐based platform combining irradiated A20 lymphoma cells with a carefully designed immunostimulatory adjuvant matrix—in both peripheral and CNSL mouse models. The results demonstrate that rWTC‐MBTA not only reduces tumor burden and prolongs survival but also induces durable, tumor‐specific immune memory.

**Figure 6:**
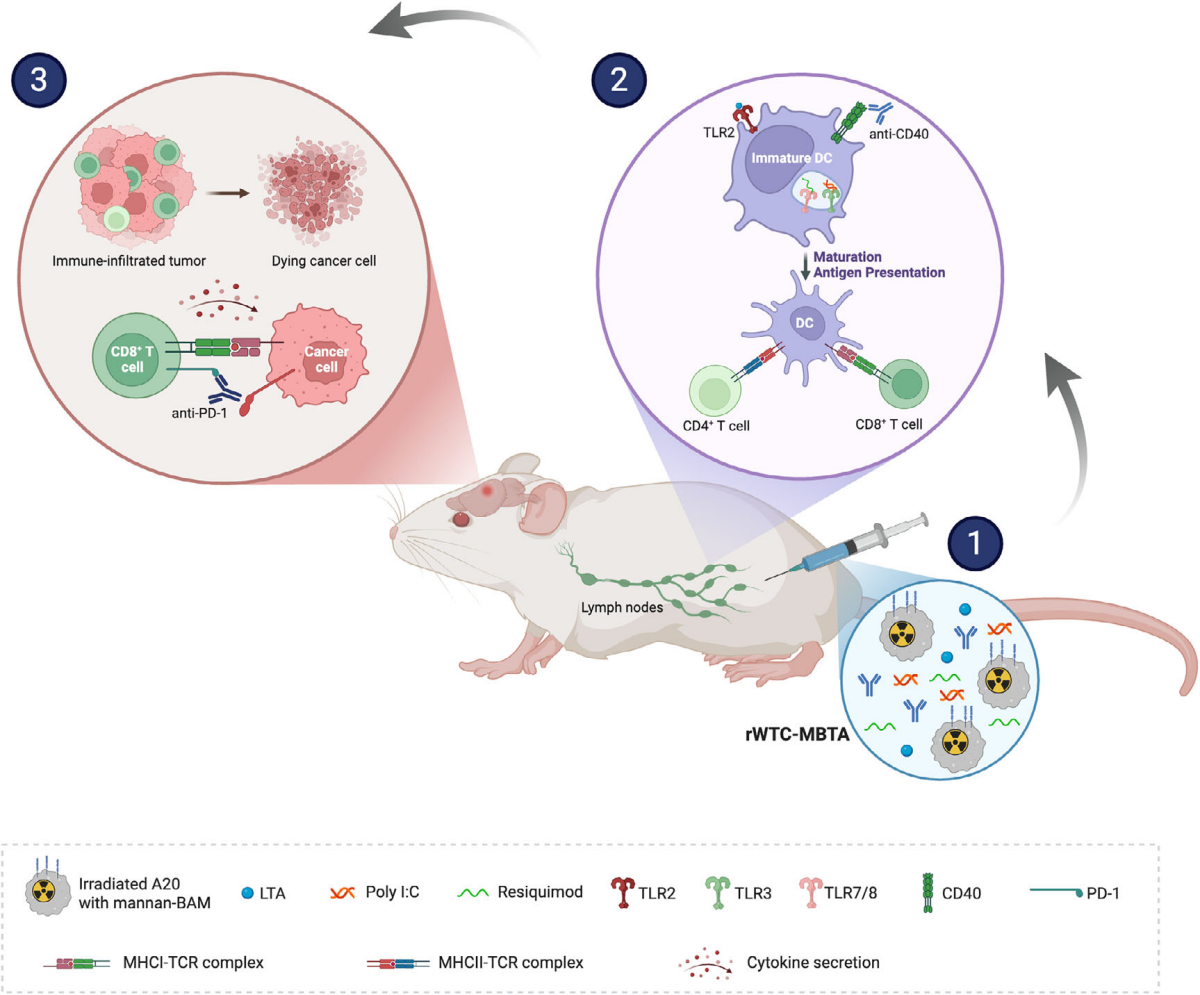
Mechanism of action of the rWTC‐MBTA vaccine in the A20 CNS lymphoma model. BALB/c mice bearing established A20 CNS lymphoma received subcutaneous rWTC‐MBTA vaccine. 1) Vaccine composition: irradiated A20 tumor cells coated with mannan‐BAM and admixed with TLR ligands—LTA (TLR2), poly I:C (TLR3), resiquimod (TLR7/8)—plus anti‐CD40 antibody. 2) Priming: TLR/CD40 signaling drives dendritic‐cell maturation and migration to draining lymph nodes, where DCs present tumor antigens and prime CD4⁺ and CD8⁺ T cells into tumor‐antigen‐specific effector and/or effector memory T cells. 3) Effector phase: Primed T cells traffic to the brain tumor and CD8⁺ T cells mediate dominant cytotoxic killing. Tumor‐infiltrating T cells upregulate PD‐1, providing a target for anti‐PD‐1 therapy to enhance and sustain antitumor activity. Created with BioRender.com.

Mechanistically, rWTC‐MBTA was designed to overcome several historical shortcomings of lymphoma vaccines (**Figure** **6**). First, the use of irradiated whole tumor cells preserves the full antigenic repertoire of the tumor, including personalized neoantigens, without requiring prior sequencing or peptide prediction.^[^ ^19^ ^]^ This contrasts with earlier idiotype vaccines like BiovaxID, which, while showing some benefit, were ultimately limited by their narrow antigenic scope and logistical complexity.^[^ ^30^, ^31^ ^]^ Second, introduction of mannan‐BAM to the tumor cell membrane enables high‐efficiency antigen uptake by dendritic cells (DCs) through C‐type lectin receptor engagement,^[^ ^32^ ^]^ which in turn promotes efficient cross‐presentation to CD8+ T cells. Third, the MBTA platform incorporates multiple Toll‐like receptor (TLR) ligands—R‐848 for TLR7/8, poly I:C for TLR3, and LTA for TLR2—thereby activating both MyD88‐ and TRIF‐dependent signaling pathways. This ensures broad innate immune activation.^[^ ^20^ ^]^ The inclusion of an anti‐CD40 antibody further licenses DCs for effective T‐cell priming, maximizing downstream adaptive immunity.

In peripheral lymphoma models, the rWTC‐MBTA vaccine induced complete tumor regression in half of the mice and conferred protection upon rechallenge after more than four months, indicative of robust immunologic memory. CD8+ T cells were essential for this effect, as demonstrated by depletion experiments. Flow cytometry revealed that vaccination significantly increased the frequency of effector and central memory T cells, along with IFN‐γ and TNF‐α production, suggesting sustained cytotoxic functionality. However, we also observed that prolonged antigen stimulation led to an upregulation of checkpoint proteins PD‐1, LAG‐3, and TIM‐3 on tumor‐infiltrating lymphocytes (TILs), as well as an increase in regulatory T cells (Tregs), indicating the development of compensatory immune regulation.

These immunosuppressive adaptations were even more prominent in the intracranial lymphoma model. Although rWTC‐MBTA vaccination delayed tumor growth, promoted parenchymal infiltration of CD8+ T cells, and doubled median survival, tumors eventually relapsed in some mice. Histopathology confirmed that the TME remained hostile, with high PD‐1 expression. These findings underscore the need for combination strategies to counteract vaccine‐induced compensatory immune regulation.

Accordingly, we tested the addition of anti‐PD‐1 with the vaccine as a combination therapy. The rationale for this approach is supported by prior studies showing that checkpoint inhibitors can reinvigorate exhausted T cells and enhance the effects of vaccination.^[^ ^33^, ^34^, ^35^ ^]^ In our models, combining rWTC‐MBTA with PD‐1 blockade not only prevented tumor recurrence but also improved overall survival in both peripheral and CNS lymphoma settings. Notably, no recurrences were observed in mice receiving the combination therapy, and survival benefits were statistically significant.

Timing of immune checkpoint inhibition relative to vaccination is also critical. Studies have shown that early administration of CTLA‐4 inhibitors enhances T‐cell priming, while PD‐1 inhibitors may be more effective when given during or after T‐cell expansion.^[^ ^36^, ^37^, ^38^ ^]^ Our two treatment schedules—concurrent and staggered anti‐PD‐1 delivery—both showed benefit, suggesting flexibility in the therapeutic window, but more detailed time‐course analyses are needed to define optimal synergy. It is also worth noting that the immune checkpoint landscape in CNS tumors is complex, and dual or sequential blockades of multiple inhibitory pathways (e.g., LAG‐3, TIM‐3, Tregs) may further improve outcomes.

Currently, no therapeutic cancer vaccine has yet been approved for lymphoma, in contrast to sipuleucel‐T for prostate cancer,^[^ ^39^ ^]^ BCG for bladder cancer,^[^ ^40^ ^]^ and T‐VEC for melanoma.^[^ ^41^ ^]^ This discrepancy reflects the immunological and logistical challenges of targeting hematologic malignancies. Lymphoma‐associated TMEs often contain immunosuppressive stromal cells, high IL‐10 levels, and few immunogenic mutations, reducing the effectiveness of traditional vaccine designs. Our study addresses these issues by combining broad antigen coverage with potent immunostimulatory adjuvants and rational combination with checkpoint blockade. The result is a system capable of priming, expanding, and maintaining tumor‐reactive T cells even within the CNS, where immune privilege and antigen loss pose serious challenges.

Looking ahead toward clinical translation, the rWTC‐MBTA vaccine platform has strong potential to eliminate minimal residual disease (MRD) in cancer therapy. Adapting the rWTC‐MBTA platform for human lymphoma would involve several key considerations. First, patient‐specific tumor sourcing would rely on diagnostic or therapeutic resections/biopsies, with feasibility varying by lymphoma subtype and accessibility of the disease site. Second, Good Manufacturing Practice (GMP)‐compliant processing, irradiation, and formulation with MBTA adjuvants will be essential, necessitating early regulatory engagement to define quality control, potency assays, and safety parameters for its multiple immunostimulatory components (Mannan‐BAM, TLR ligands, and anti‐CD40 antibody). Third, rapid manufacturing timelines are critical for aggressive lymphomas. Turnaround time can be improved by prospective cryopreservation of patient‐derived tumor cells at diagnosis and pre‐arranged logistics; because lots are individualized, decentralized/regional GMP capacity with validated cold‐chain and process automation will aid scalability. Finally, clinical immunobiology must be carefully considered, as lymphoma subtypes differ in baseline immunogenicity. Consistent with our “hot” versus “cold” tumor model observations, certain subtypes may respond adequately to simplified adjuvant combinations, whereas others may require the full MBTA formulation to overcome immune suppression. Together, these considerations highlight both the feasibility and key challenges of translating the rWTC‐MBTA platform into a personalized vaccine approach for human lymphoma.

In conclusion, our results demonstrate that rWTC‐MBTA is a potent immunotherapeutic platform capable of inducing tumor‐specific immunity, delaying progression, and synergizing with PD‐1 inhibition to improve outcomes in both peripheral and CNS lymphoma. By overcoming the intrinsic limitations of prior vaccine strategies—including restricted antigen breadth, weak immunostimulation, and poor CNS delivery—this platform offers a promising foundation for next‐generation vaccine‐based immunotherapy in lymphoma. Further refinement and clinical translation will be necessary, but these findings offer compelling preclinical support for continued development.

## 4 Experimental Section

### Reagents, Cell Lines, and Animals

*Reagents*: Mannan, derived from *Saccharomyces cerevisiae*, along with polyinosinic‐polycytidylic (poly I:C) acid sodium salt and lipoteichoic acid from *Bacillus subtilis*, were obtained from Sigma–Aldrich (St. Louis, MO). Resiquimod (R‐848) was sourced from Tocris Bioscience (Minneapolis, MN). Biocompatibility Anchor for Cell Membranes (BAM) was obtained from NOF America (White Plains, NY). Antibodies, including anti‐mouse CD40 (clone FGK4.5), anti‐mouse CD4 (clone GK1.5), and anti‐mouse CD8 (clone 53‐6.7), were acquired from BioXCell (West Lebanon, NH). Anti‐mouse CD279 (PD‐1) (clone RMP1‐14) was purchased from Leinco Technologies, Inc. (Fenton, MO).

*Cell Line*: The A20 murine lymphoma cell line was obtained from the American Type Culture Collection (ATCC). B‐luc‐A20 cell line was purchased from Biocytogen. Both cell lines were maintained in RPMI 1640 medium, supplemented with 10% (v/v) fetal bovine serum (FBS) and 1% penicillin/streptomycin (Gibco, Gaithersburg, MD).

*Animals*: BALB/c mice (6–8 weeks) were purchased from Charles River Laboratories (Wilmington, MA). All procedures involving animals in this study, conducted by staff affiliated with the NCI Center for Cancer Research (CCR), received approval (ASP: NOB‐010) from the NCI Animal Care and Use Committee (ACUC). These procedures adhered to federal regulations and standards. The intramural NIH Animal Care and Use (ACU) program, where this research was carried out, is accredited by AAALAC International.

### Animal Experiments

*Syngeneic Peripheral Lymphoma Model*: To establish the subcutaneous A20 lymphoma model, BALB/c mice were injected subcutaneously in the right flank with 1 million A20 cells suspended in 100 µL of PBS. On day 11 post‐injection, when the mean tumor volume reached ≈60 mm^3^, mice were randomized into various treatment groups. The vaccine was administered subcutaneously to the left flank for three consecutive days each week over a four‐week period. Tumor growth was monitored every other day, with tumor volumes measured using calipers and calculated using the formula V = ½ (Length × Width^2^). Survival endpoints were defined as either a tumor volume of 2000 mm^3^ or a tumor length of 2 cm, at which point survival time was recorded for analysis.

For immune response analysis across different sites and time points, a dynamic study of peripheral lymphomas was conducted. Five mice per group were euthanized to collect tumor‐draining lymph nodes (tdLN), contralateral injection‐site lymph nodes (isLN), and spleens on day 14 (after the first treatment cycle, days 11‐13) and day 21 (after the second treatment cycle, days 18–20). Those samples were analyzed via histopathology, flow cytometry, and co‐culture experiments. For the re‐challenge experiment, mice that achieved complete remission for 150 days, along with naïve female BALB/c mice, were inoculated with 1 million A20 cells in the left flank, opposite to the site of primary inoculation. In the T‐cell depletion model, mice were injected with 250 µg of anti‐CD4 antibody (clone GK1.5; BioXcell) and/or 250 µg of anti‐CD8 antibody (clone 53‐6.7; BioXcell) on Day ‐2, ‐1, the day of A20 cell injection, and weekly thereafter. The mice were divided into five groups: control, rWTC‐MBTA vaccinated, CD4‐depleted, CD8‐depleted, and both CD4‐ and CD8‐depleted. All groups, except the control, received the rWTC‐MBTA vaccine treatment.

*Syngeneic CNS Lymphoma Model*: To establish the CNS lymphoma model, female BALB/c mice were intracranially injected with 50 000 A20 or 100 000 B‐luc‐A20 cells suspended in 2 µL of Hank's Balanced Salt Solution (HBSS; Crystalgen) at 1 mm rostral to the bregma, 2 mm to the right of the midline, and 2 mm deep from the skull surface. Mice were monitored every three days initially, with daily observations beginning two weeks post‐injection. Survival endpoints were based on any of the following criteria: >15% loss in initial body weight, visible tumor protrusion from the skull, significant hunched posture with decreased mobility, or ataxia. The rWTC‐MBTA vaccine was administered subcutaneously to the flank for three consecutive days each week over four weeks, starting five days after intracranial injections. For dynamic studies, three mice per group were euthanized on days 8, 15, and 22 post‐inoculation, and their brains were collected for subsequent analysis.

### Flow Cytometry

Approximately 500 000 cells were used for each panel. Detailed antibody information for all flow cytometry staining panels is provided in the Supplementary Table (Table S1, Supporting Information). Dead cells were identified by staining with a live/dead fixable dye (ThermoFisher Scientific, Cat No. L23105) in PBS at room temperature for 15 minutes. Cells were then incubated with TruStain FcX PLUS (anti‐mouse CD16/32, BioLegend, Cat No. 156604) at 4 °C for 20 min to block Fc receptors. Surface marker staining was performed in flow staining buffer (PBS with 2% FBS, 1 mm EDTA, and 0.02% sodium azide) at 4 °C for 30 min. For intracellular FOXP3 staining, the eBioscience Foxp3/Transcription Factor Staining Buffer Set (ThermoFisher Scientific, Cat No. 00‐5523‐00) was used following surface marker staining. Stained cells were analyzed on a BD FACSymphony A5 flow cytometer, and data were processed using FlowJo software (version 10.10.0).

Dendritic cells (DCs) were defined by the markers CD45+/Dump−/F4/80−/CD11c+/MHCII+. Within the DC population, conventional DCs (cDCs) were characterized by Ly6C− expression, while monocyte‐derived DCs (moDCs) were identified as Ly6C+/CD11b+. Plasmacytoid DCs (pDCs) were identified as Ly6C+/CD11b−. Among cDCs, cDC1 was distinguished by SIRPα−/XCR1+ expression, which included migratory cDC1 (CD103+) and resident cDC1 (CD8α+). cDC2 were identified by SIRPα+/XCR1− expression (Figure S7, Supporting Information). Naive T cells were defined as CD44−/CD62L+, central memory (CM) T cells as CD44+/CD62L+, and effector and/or effector memory (E/EM) T cells as CD44+/CD62L−. Gating schematics for innate and adaptive immune subsets are provided in Supporting figures (Figures S7 and S8, Supporting Information). IFN‐γ and TNF‐α in the supernatants of tumor cells co‐cultured with immune cells were analyzed using the BD CBA Mouse Inflammation Kit (BD Biosciences, Cat No. 552364) via flow cytometry.

### Vaccine Preparation

To prepare the rWTC‐MBTA vaccine, well‐cultured A20 or B‐luc‐A20 lymphoma cells were collected and resuspended at a density of 1 million cells in 50 µL of PBS per mouse dose.^[^ ^18^ ^]^ The cells were irradiated with 100 Gy using a 137Cs MARK I model irradiator (JL Shepherd & Associates, San Fernando, CA). The MBTA solution, containing 0.2 mm Mannan‐BAM, 25 µg resiquimod (R‐848), 25 µg polyinosinic‐polycytidylic acid (poly(I:C)), 25 µg lipoteichoic acid (LTA), and 20 µg anti‐CD40 antibody, was prepared following previous reports.^[^ ^18^ ^]^ Irradiated tumor cells (1 million A20 cells in 50 µL PBS) were incubated with 50 µL of MBTA solution for 30 min to 1 h on ice. The resulting rWTC‐MBTA vaccine (100 µL per mouse) was administered subcutaneously into the flank region of the animals according to the treatment schedule.

### Tissue Dissociation

Lymph nodes and spleens were collected and processed into single‐cell suspensions by grinding and filtering through a 70 µm MACS SmartStrainer (Miltenyi Biotec, Inc., Gaithersburg, MD). Tumor tissues were isolated and digested using a mouse Tumor Dissociation Kit and a GentleMACS Dissociator (Miltenyi Biotec, Inc., Gaithersburg, MD) according to the manufacturer's instructions. Following tissue dissociation, erythrocytes were removed using 1× RBC lysis buffer.

### Histology Staining

For hematoxylin and eosin (H&E) staining, mouse brains were collected at designated time points, fixed in 10% buffered formalin, and embedded in paraffin. Sections (4 µm thick) were deparaffinized, rehydrated, stained with hematoxylin for nuclei, and counterstained with eosin to visualize cytoplasm. For immunohistochemistry (IHC) staining, tissue sections underwent deparaffinization, rehydration, and antigen retrieval. Non‐specific binding was blocked before overnight incubation at 4 °C with primary antibodies, including anti‐CD19, anti‐CD4, anti‐CD8, and anti‐PD1. Detection was performed using MACH4 HRP‐Polymer, followed by DAB staining and hematoxylin counterstaining (Biocare Medical, CA, USA).

### Adoptive Cell Transfer

To assess whether vaccine‐primed lymphocytes can access CNS tumors, injection‐site lymph nodes (isLNs) were harvested from donor mice after three weeks of rWTC‐MBTA vaccination (prepared with B‐luc–A20 cells). Single‐cell suspensions were labeled with CFSE (10 µm in PBS, 15 min, 37 °C). ≈2 million viable cells in 100 µL PBS were injected intravenously (tail vein) into B‐luc–A20 CNS lymphoma recipient mice on day 26 after intracranial tumor inoculation. Three days later, recipient mice were transcardially perfused with cold PBS to remove intravascular cells. Brains were then dissociated, and brain‐infiltrating leukocytes were isolated according to the published protocol.^[^ ^22^ ^]^ The flow cytometry staining panel for CFSE⁺ donor cells was included in the Table S1 (Supporting Information).

### Statistical Analysis

Statistical analyses were performed in GraphPad Prism (v10.2.0, GraphPad Software, San Diego, CA), except that Cox proportional hazards regression was conducted in SPSS. Comparisons between two groups used unpaired t tests, and comparisons across more than two groups used one‐way ANOVA. Longitudinal BLI was analyzed by two‐way ANOVA (treatment × time) on log10‐transformed photon flux. Survival was evaluated by Kaplan–Meier with the log‐rank (Mantel–Cox) test. Unless noted, data are presented as mean ± SD (BLI, mean ± SEM). *p* < 0.05 was considered statistically significant.

The authors would like to thank the staff of the NCI‐CCR‐affiliated animal facility for their support with animal housing and monitoring, the NCI CCR Flow Cytometry Core for their expertise and support in flow cytometry, and the NCI CCR LCBG Microscopy Core for scanning the histology slides. This research was supported by the Intramural Research Program of the NIH, National Cancer Institute (NCI, ZIA BC 011773, to Z.Z.), and the Eunice Kennedy Shriver National Institute of Child Health and Human Development (NICHD). This study was partially conducted under a CRADA research agreement with NE1 Inc.

## Conflict of Interest

Dr. Samik Chakraborty is employed by NE1 Inc. Dr. Herui Wang and Dr. Zhengping Zhuang collaborate with NE1 Inc. under CRADA. Dr. Karel Pacak is the Scientific Advisor to NE1 Inc.

## Author Contributions

Y.Z. and J.Y. contributed equally to this work. Y.Z., H.W., Z.Z., W.S., and K.P. designed research. Y.Z., H.W., J.Y., M.S., Samik.C., A.V., Shuran.C., and Q.X. performed research. Y.Z., J.Y., H.W., and Z.Z. analyzed data. Y.Z. wrote the first draft of the manuscript. M.S., Samik C., W.S., K.P., H.W., and Z.Z. reviewed and revised the paper. H.W. and Z.Z. supervised this study.

## Supporting information



Supporting Information

Supporting Information

## Data Availability

The data that support the findings of this study are available on request from the corresponding author. The data are not publicly available due to privacy or ethical restrictions.
