# Supplementary material for: rWTC‐MBTA Vaccine, Alone and Enhanced with Anti‐PD1, Elicits Immune Responses against CNS and Peripheral B‐Cell Lymphoma
Source: Adv Sci (Weinh). 2025 Dec 8;13(35):e11605. doi: 10.1002/advs.202511605 (PMC13292266; doi:10.1002/advs.202511605)
Supplement: Supplementary file 1 — Supporting Information [file ADVS-13-e11605-s001.docx]

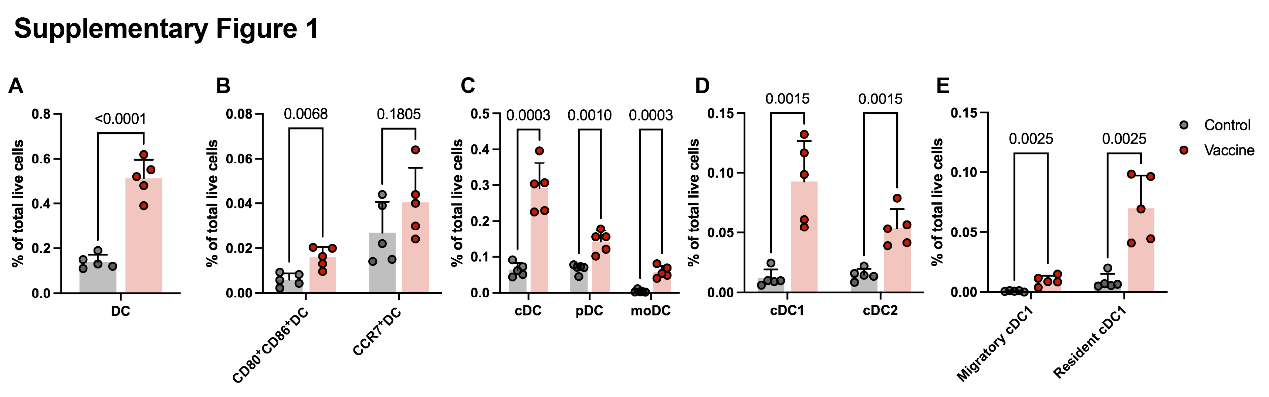


**Supplementary Figure 1. Percentage of total DC (A) and DC subset (B-E) in total live cells after the 2nd vaccination cycle.** N=5 for each group; unpaired T-test.


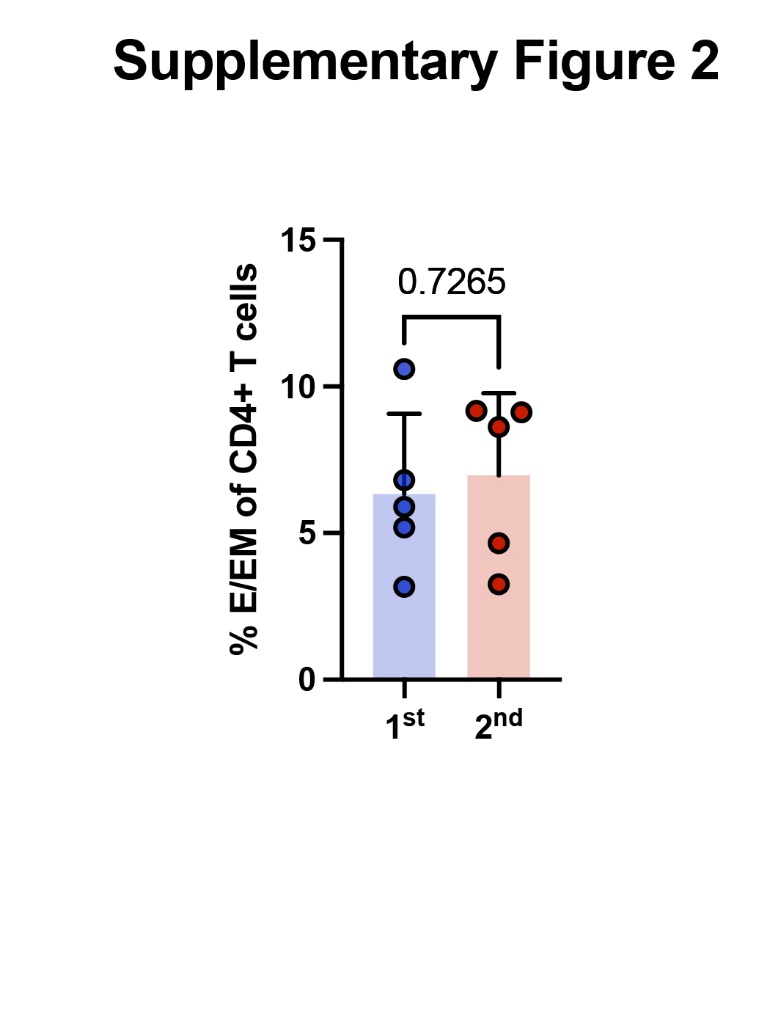


**Supplementary Figure 2. Effector and/or effector memory (E/EM) CD4+ T cell frequencies remain stable during sequential immunizations.**

Frequencies of effector and/or effector memory (E/EM) CD4+ T cells in lymph nodes showed no significant change following the second tumor vaccination cycle compared to levels observed after the first vaccination. N=5 for each group; *P*=0.7265, unpaired T-test.


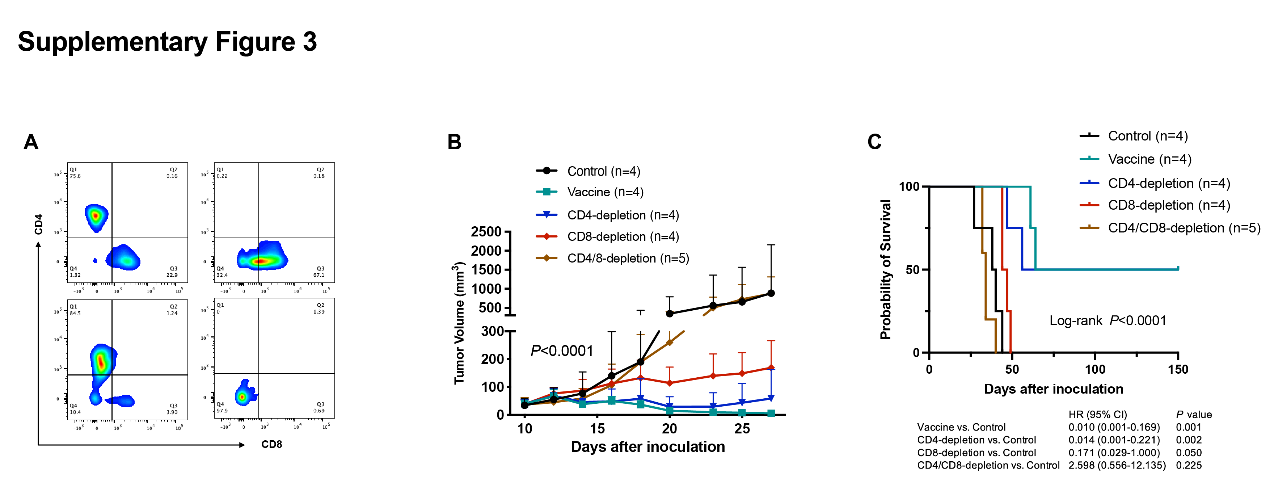


**Supplementary Figure 3. T-cell depletion experiments demonstrate T cell-dependent antitumor efficacy of the rWTC-MBTA vaccine.**

(A) Flow cytometry confirmation of CD4^+^ T cell, CD8^+^ T cell, or dual CD4^+^/CD8^+^ T cell depletion in peripheral blood mononuclear cells. (B) Tumor growth kinetics, expressed as mean tumor volume ± SEM, in mice treated with rWTC-MBTA vaccine under T cell-depleted or control conditions. Statistical significance was determined by one-way ANOVA (*P*<0.0001). (C) Kaplan-Meier survival curves comparing survival outcomes between T cell-depleted and control groups. Survival differences were analyzed using the log-rank (Mantel-Cox) test (*P*<0.0001). Hazard ratios with 95% CI are also shown.


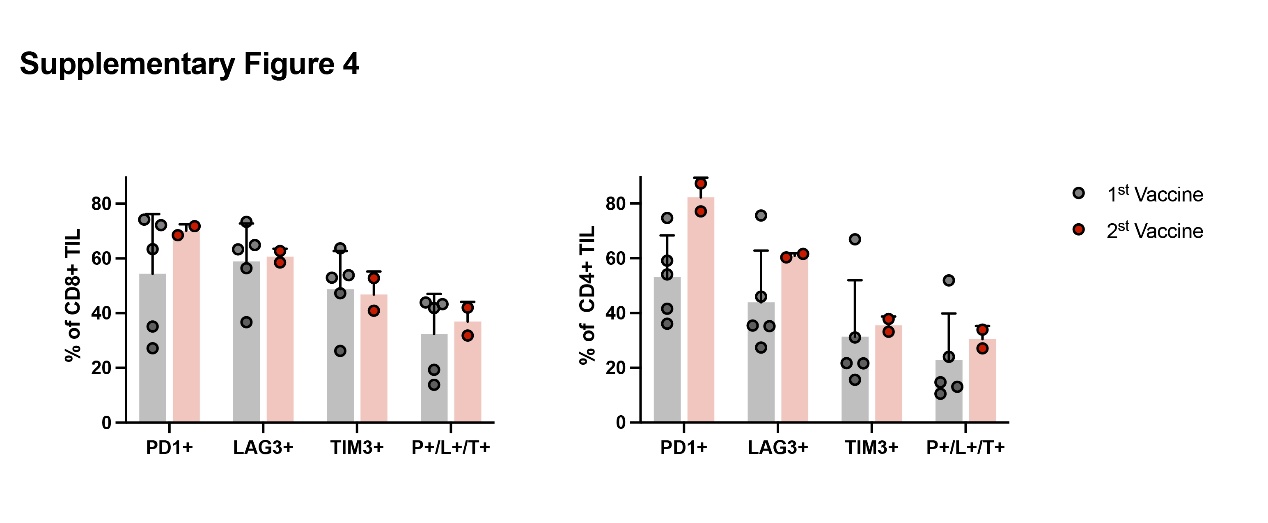


**Supplementary Figure 4. The exhaustion marker expression in tumor-infiltrating CD8^+^ and CD4^+^ T cells was stable between sequential rWTC-MBTA vaccine cycles.**

Expression of immune checkpoint markers (PD-1, LAG-3, TIM-3) associated with T cell checkpoint molecules was evaluated on tumor-infiltrating CD8^+^ and CD4^+^ T cells after the first and second vaccine cycles.


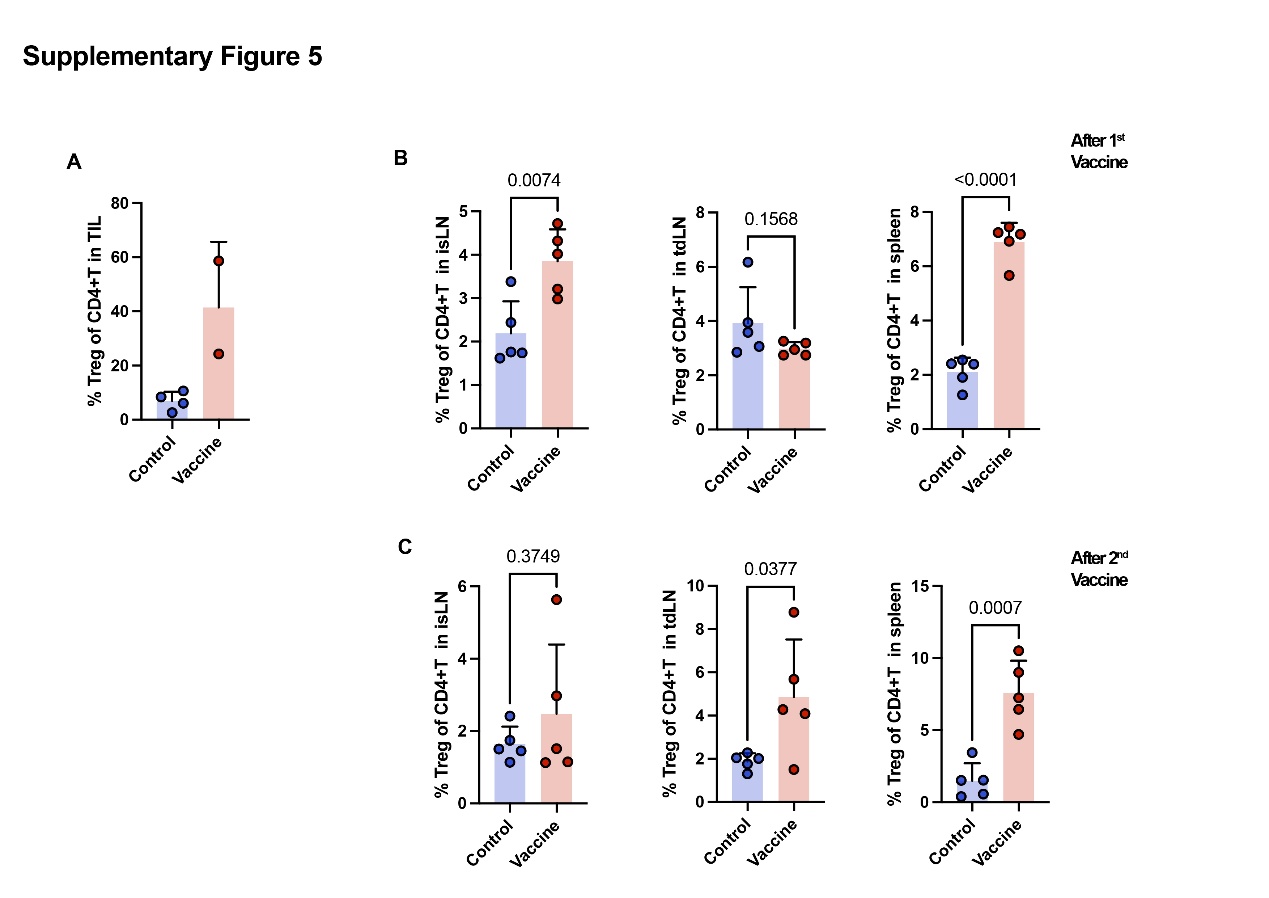


**Supplementary Figure 5. The rWTC-MBTA vaccine increases regulatory T cells in tumor and lymphoid tissues.**

**(A)** Increased proportions of Tregs in the tumor microenvironment of vaccinated mice (n=2) compared to controls (n=4). **(B-C)** Elevated Treg frequencies in lymphatic tissues, with the spleen showing the most pronounced expansion. N=5 for each group; unpaired T-test.


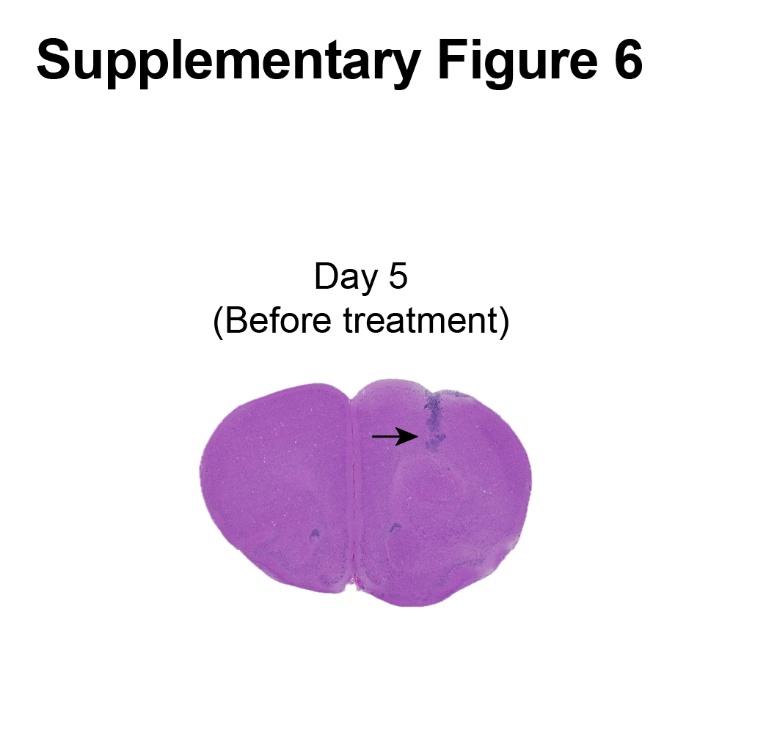


**Supplementary Figure 6. Establishment of an orthotopic CNS lymphoma mouse model for rWTC-MBTA vaccine evaluation.**

Representative HE-stained brain sections 5 days post-inoculation, showing successful tumor engraftment. Black arrow: invasive lymphoma clusters.


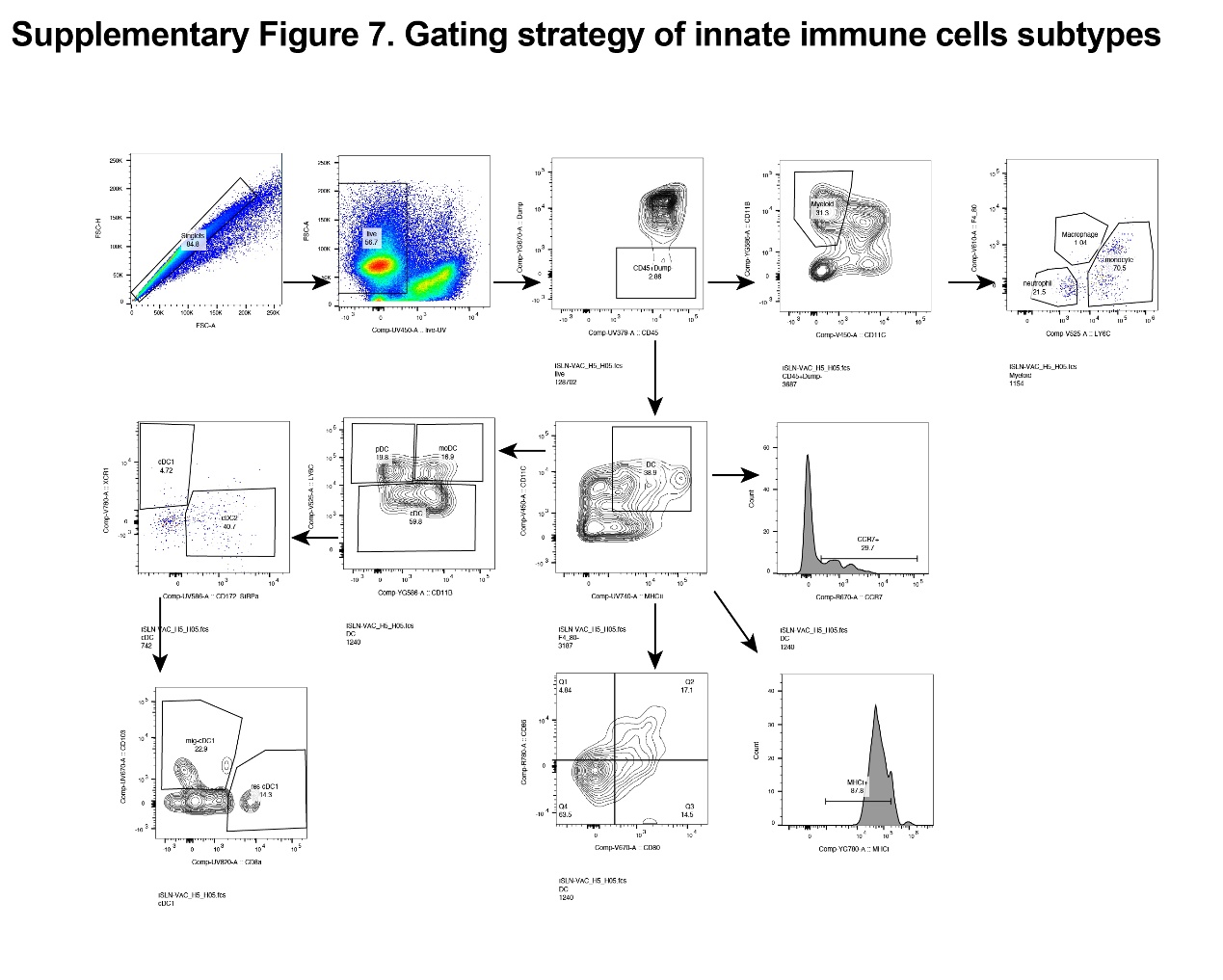


**Supplementary Figure 7. Gating strategy of dendritic cell subtypes.**


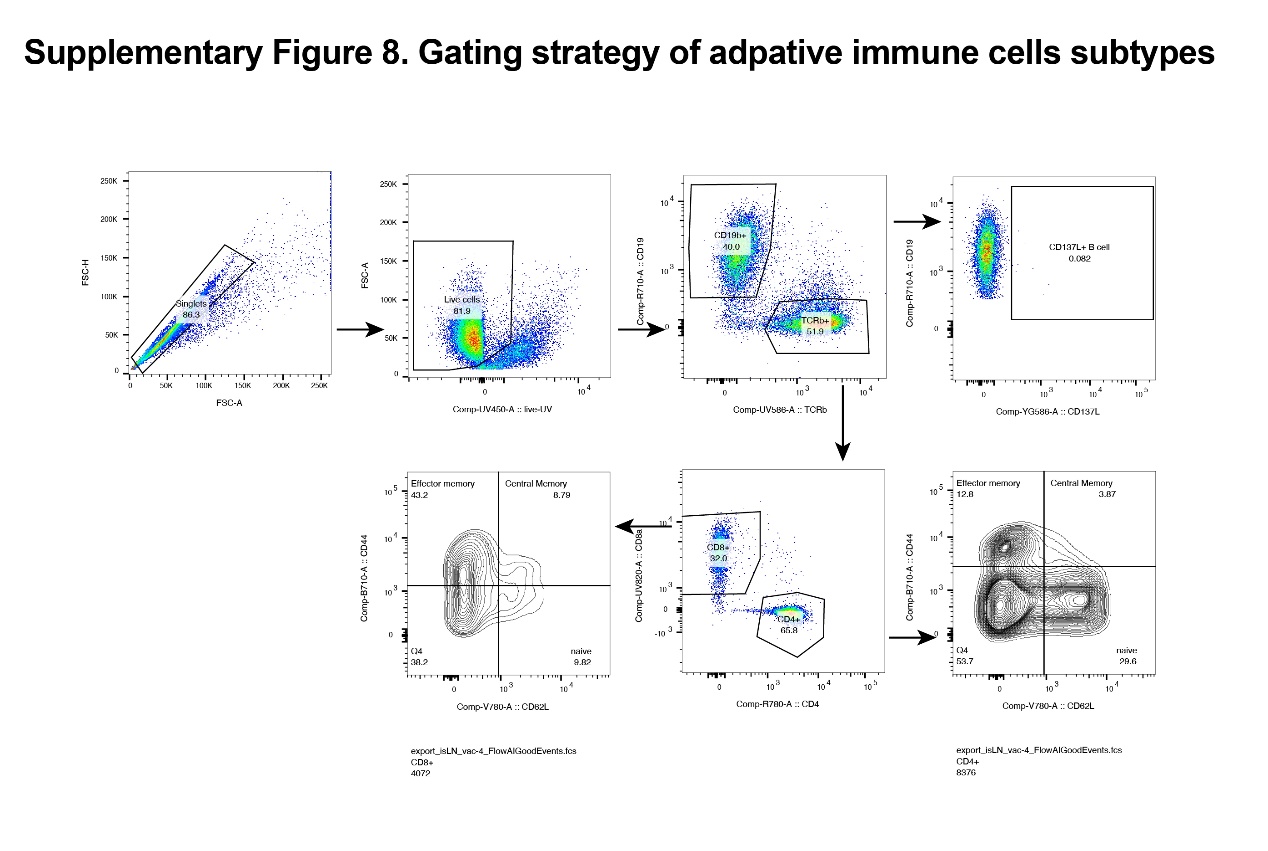


**Supplementary Figure 8. Gating strategy of adaptive immune subsets.**

**Supplementary Table 1. Flow staining panel used in this study.**
